# Transcriptomic analysis of human cartilage identified potential therapeutic targets for hip osteoarthritis

**DOI:** 10.1093/hmg/ddae200

**Published:** 2025-01-08

**Authors:** Jingyi Huang, Ming Liu, Andrew Furey, Proton Rahman, Guangju Zhai

**Affiliations:** Human Genetics & Genomics, Division of BioMedical Sciences, Faculty of Medicine, Memorial University of Newfoundland, 300 Prince Philip Drive, St. John’s, Newfoundland & Labrador, A1B 3V6, Canada; Human Genetics & Genomics, Division of BioMedical Sciences, Faculty of Medicine, Memorial University of Newfoundland, 300 Prince Philip Drive, St. John’s, Newfoundland & Labrador, A1B 3V6, Canada; Discipline of Orthopaedic Surgery, Faculty of Medicine, Memorial University of Newfoundland, 300 Prince Philip Drive, St. John’s, Newfoundland & Labrador, Canada A1B 3V6 & Office of the Premier, Government of Newfoundland & Labrador, 100 Prince Philip Drive, St. John's, Newfoundland & Labrador, A1B 4J6, Canada; Discipline of Medicine, Faculty of Medicine, Memorial University of Newfoundland, 300 Prince Philip Drive, St. John's, Newfoundland & Labrador, A1B 3V6, Canada; Human Genetics & Genomics, Division of BioMedical Sciences, Faculty of Medicine, Memorial University of Newfoundland, 300 Prince Philip Drive, St. John’s, Newfoundland & Labrador, A1B 3V6, Canada

**Keywords:** Osteoarthritis, hip, transcriptomics, gene expression

## Abstract

Cartilage degradation is the hallmark of osteoarthritis (OA). The purpose of this study was to identify and validate differentially expressed genes (DEGs) in human articular cartilage that could serve as potential therapeutic targets for hip OA. We performed transcriptomic profiling in a discovery cohort (12 OA-free and 72 hip OA-affected cartilage) and identified 179 DEGs between OA-free and OA-affected cartilage after correcting for multiple testing (*P* < 2.97 × 10^−6^). Pathway and network analyses found eight hub genes to be associated with hip OA (*ASPN*, *COL1A2*, *MXRA5*, *P3H1*, *PCOLCE*, *SDC1*, *SPARC*, and *TLR2*), which were all confirmed using qPCR in a validation cohort (36 OA-free and 62 hip OA-affected cartilage) (*P* < 6.25 × 10^−3^). Our data showed that dysregulation of extracellular matrix formation and imbalance in the proportion of collagen chains may contribute to the development of hip OA, and *SDC1* could be a promising potential therapeutic target. These findings provided a better understanding of the molecular mechanisms for hip OA and may assist in developing targeted treatment strategies.

## Introduction

Osteoarthritis (OA) is a degenerative joint disease characterized by the progressive degradation of articular cartilage [1]. It is one of the most common causes of disability in the world, severely affecting quality of life [2]. Age, sex, obesity, joint injury, structural and development abnormalities, and genetic factors are well-recognized risk factors for OA [3], and hip OA has the highest heritability estimate from twins and family studies [4]. However, the underlying molecular mechanisms for hip OA development remain incompletely understood [5].

Previous studies have highlighted several altered pathways involved in OA, such as inflammation, extracellular matrix (ECM) remodeling, and cell death [6–8]. Aigner T. *et al.* applied transcriptomic analysis to cartilage and identified continuous oxidative stress in cells and matrix mediated by matrix metalloproteinases family and oxidative defense genes as key factors in the pathogenesis of knee OA [9, 10]. Karlsson C. *et al.* revealed that the expression levels of genes encoding collagen (*COL13A1*, *COL14A1*, *COL15A1*, and *COL8A2*) were significantly higher in OA-affected cartilage than in OA-free cartilage [11]. Aki T. *et al.* studied secondary hip OA and identified a number of genes associated with ECM-receptor interaction [12]. However, transcriptomic analyses often identify hundreds to thousands of significantly differentially expressed genes (DEGs) with complex and interrelated regulatory interactions between them [13]. Therefore, identifying hub genes is essential for understanding key regulatory pathways because hub gene locates at central position within gene regulatory network or a protein–protein interaction network [14]. These genes typically act as central nodes in biological pathways, and changes in their expression can lead to pathway dysregulation or disease. On the other hand, most of these studies did not clearly point out the etiology of their OA participants, such as primary, secondary, or post-traumatic, or did not differentiate the affected joints. Transcriptomic studies focused on primary hip OA are still scarce. The lack of detailed analysis hinders our understanding of the molecular mechanisms that differentiate primary hip OA from other types of OA. Therefore, more targeted studies in this area are urgently needed to develop effective diagnostic and therapeutic strategies. Furthermore, the Newfoundland & Labrador (NL) population has the highest prevalence of OA in Canada and has a unique genetic structure due to historical and geographic isolation [15], which provides us an increased power to identify genetic factors not only for monogenic disorder but also for complex traits [16]. We leveraged this opportunity from the NL OA population and performed a transcriptomic analysis of human cartilage for hip OA.

## Results

### Participants’ demographic factors

A total of 72 primary hip OA patients and 12 OA-free controls were included in the discovery cohort, with hip OA patients being significantly younger and having a higher body mass index (BMI) than the controls (*P* = 1.47 × 10^−4^ and 0.01, respectively). The validation cohort included 62 primary hip OA patients and 36 OA-free controls, with significant differences in age, sex, and BMI between the hip OA patients and the controls. Between the discovery and validation cohorts, these demographic factors were similar in the hip OA patients as well as in the controls. More details are presented in Table 1.

**Table 1:** Comparison of demographic factors between hip OA patients and OA-free controls in discovery cohort and validation cohort.

	**Discovery cohort**		**Validation cohort**		**P** ^**1**^	**P** ^**2**^	**P** ^**3**^	**P** ^**4**^
	**Hip OA** **N = 72**	**OA-free control** **N = 12**	**Hip OA** **N = 62**	**OA-free control** **N = 36**				
Age (yrs)	67.8 ± 10.6	82.8 ± 10.0	67.4 ± 10.1	79.5 ± 9.4	1.47 × 10^−4^	4.1 × 10^−8^	0.80	0.31
BMI (kg/m^2^)	30.8 ± 6.2	23.3 ± 4.9	31.8 ± 5.9	25.1 ± 4.3	0.01	8.0 × 10^−5^	0.46	0.48
Sex (N, female)	40	9	29	30	0.34	8.1 × 10^−4^	0.71	0.83

Values are either mean ± standard deviation or number. *P*-values were obtained by Wilcoxon signed-rank test (age, BMI) or Chi-squared test (sex). P^1^: statistical differences between hip OA and OA-free control in discovery cohort; P^2^: statistical differences between hip OA and OA-free control in validation cohort; P^3^: statistical differences between hip OA in discovery cohort and in validation cohort; P^4^: statistical differences between OA-free control in discovery cohort and in validation cohort. BMI: body mass index; OA: osteoarthritis.

### Identification of hub genes

Thirty-one OA-affected cartilage and five OA-free cartilage samples were sequenced with RNA-Seq, while 44 OA-affected cartilage and seven OA-free cartilage samples were profiled with the microarray approach. Three samples were assayed using both approaches to assess the concordance between the results obtained using these two approaches. A total of 32 476 genes were detected by the two approaches, and after removing genes expressed in less than 80% of the samples, 16 831 genes remained. In a previous study comparing cross-platform normalization methods, the Z-score transformation demonstrated a Kappa value of approximately 0.75, a parameter indicative of interrater reliability [17]. We therefore applied Z-score transformation to both approaches before combining the two datasets. The three samples assayed using both methods demonstrated excellent concordance between the results obtained by the two approaches, and the Wilcoxon signed-rank test showed that no gene showed significant differences (all *P* > 0.05).

**Figure 1:**
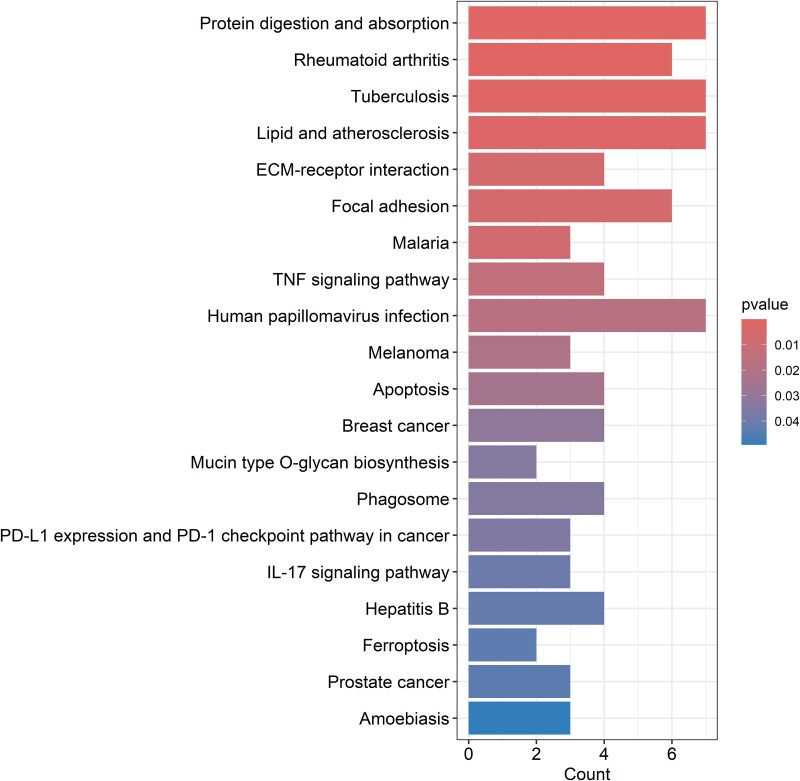
Kyoto Encyclopedia of genes and genomes (KEGG) analyses results of 179 differentially expressed genes.

**Figure 2:**
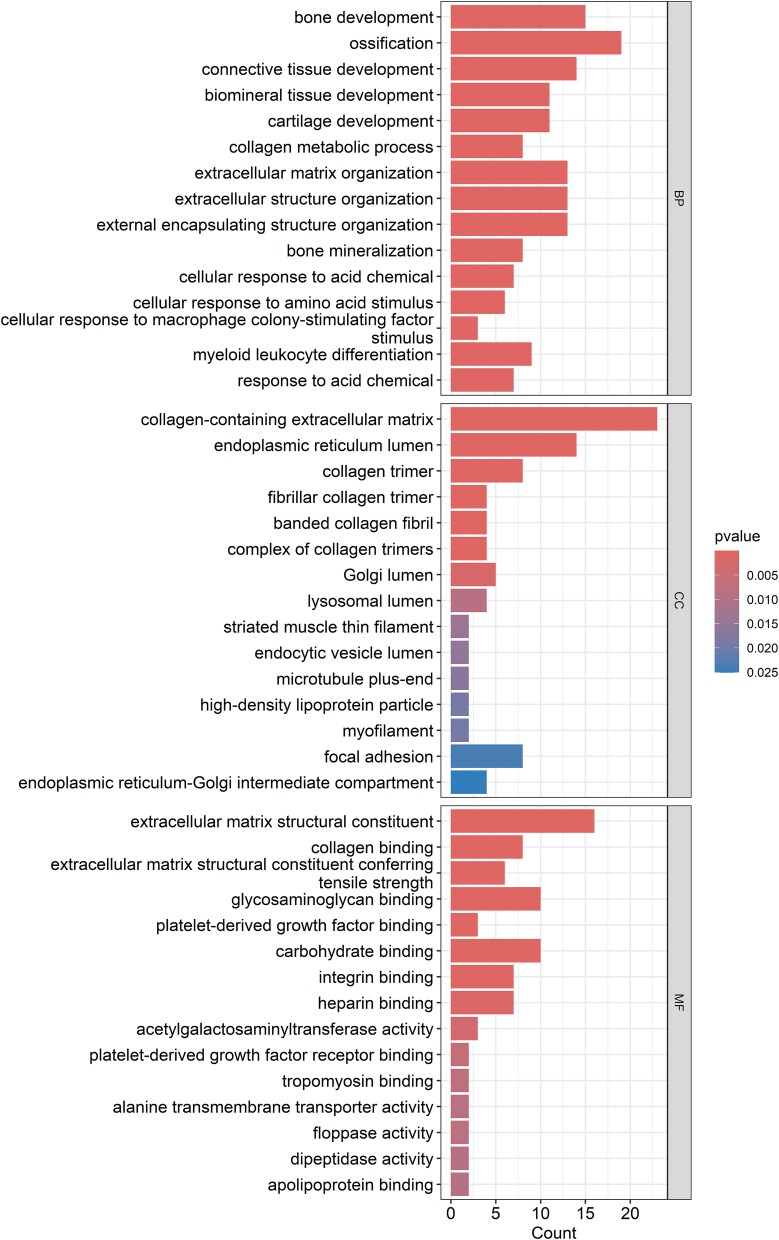
Gene ontology (GO) analyses results of 179 differentially expressed genes.

179 DEGs between OA-affected cartilage and OA-free cartilage were identified (Full list of DEGs was provided in Supplementary Table S1) (*P* < 2.97 × 10^−6^, after Bonferroni correction for 16 831 genes) [18]. The KEGG pathway analysis revealed that the DEGs were enriched in the protein digestion and absorption (*P* = 1.76 × 10^−5^), rheumatoid arthritis (*P* = 9.76 × 10^−5^), lipid and atherosclerosis (*P* = 1.66 × 10^−3^), and ECM-receptor interaction (*P* = 5.68 × 10^−3^) pathways (Fig. 1). The GO enrichment analysis revealed that the DEGs were significantly enriched in bone development (*P* = 4.54 × 10^−10^), ossification (*P* = 7.59 × 10^−9^), connective tissue development (*P* = 1.50 × 10^−7^), and cartilage development (*P* = 1.58 × 10^−6^) in BP domain; collagen-containing extracellular matrix (*P* = 1.50 × 10^−7^), collagen trimer (*P* = 5.54 × 10^−7^), and fibrillar collagen trimer (*P* = 2.10 × 10^−6^) in CC domain; and extracellular matrix structural constituent (*P* = 9.33 × 10^−13^) and collagen binding (*P* = 1.14 × 10^−7^) in MF domain (Fig. 2).

**Figure 3:**
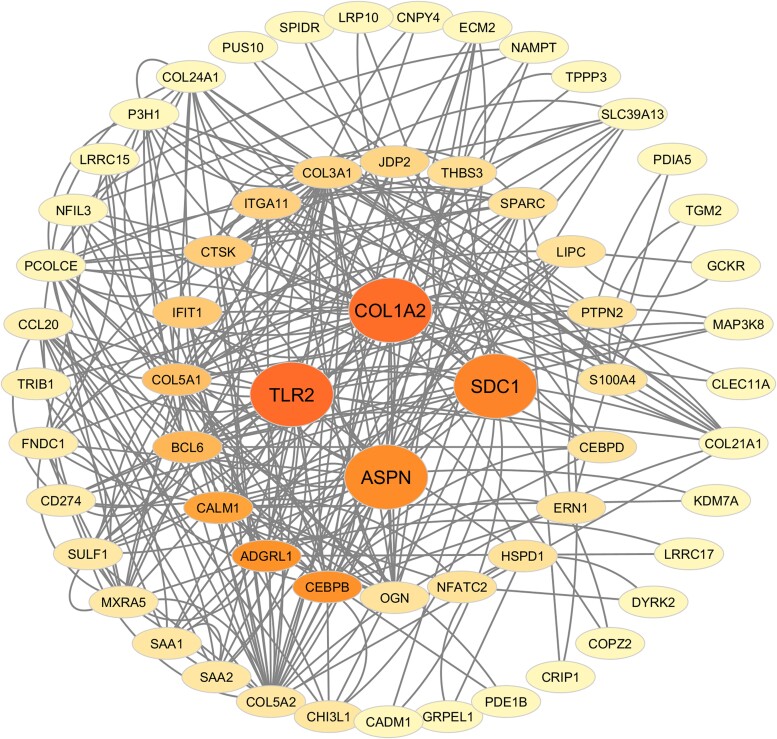
Protein–protein interaction network of 179 differentially expressed genes. The protein–protein interaction network was sorted by betweenness centrality score.

**Figure 4:**
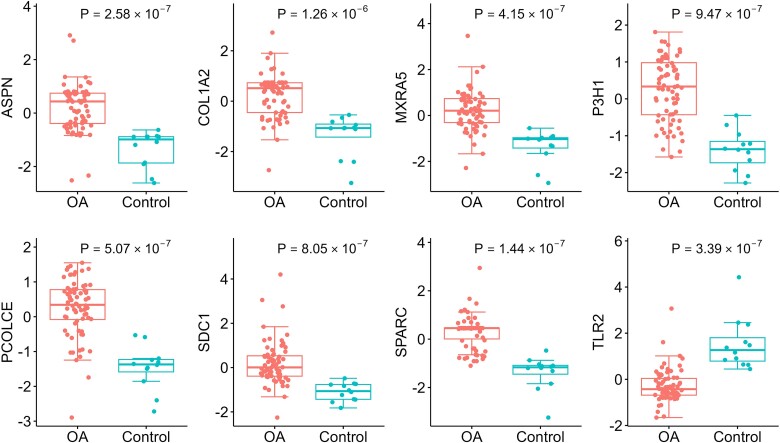
Boxplots of the expression levels of the eight hub genes in discovery cohort. OA: Osteoarthritis; *ASPN*: Asporin; *COL1A2*: Collagen type I alpha 2 chain; *MXRA5*: Matrix remodeling associated 5; *P3H1*: Prolyl 3-hydroxylase 1; *PCOLCE*: Procollagen C-endopeptidase enhancer; *SDC1*: Syndecan 1; *SPARC*: Secreted protein acidic and cysteine rich; *TLR2*: Toll-like receptor 2.

Furthermore, the STRING analysis constructed a network with 170 nodes, 226 edges, an average node degree of 2.66, and a PPIs enrichment *P*-value of < 1.0 × 10^−16^, indicating biologically relevant and potentially functionally interconnected protein interactions. Asporin (*ASPN*), collagen type I alpha 2 chain (*COL1A2*), matrix remodeling associated 5 (*MXRA5*), prolyl 3-hydroxylase 1 (*P3H1*), procollagen C-endopeptidase enhancer (*PCOLCE*), syndecan 1 (*SDC1*), secreted protein acidic and cysteine rich (*SPARC*), and toll-like receptor 2 (*TLR2*) were identified as hub genes by cross-comparing the betweenness, closeness, degree, DMNC, MCC, and MNC scores obtained from the CytoHubba plug-in (Fig. 3). Among them, seven genes (*ASPN*, *COL1A2*, *MXRA5*, *P3H1*, *PCOLCE*, *SDC1*, and *SPARC*) showed significantly higher expression levels in OA-affected cartilage than in OA-free cartilage in discovery cohort, while *TLR2* exhibited lower expression level in OA-affected cartilage (Fig. 4).

### Validation of hub genes

**Figure 5:**
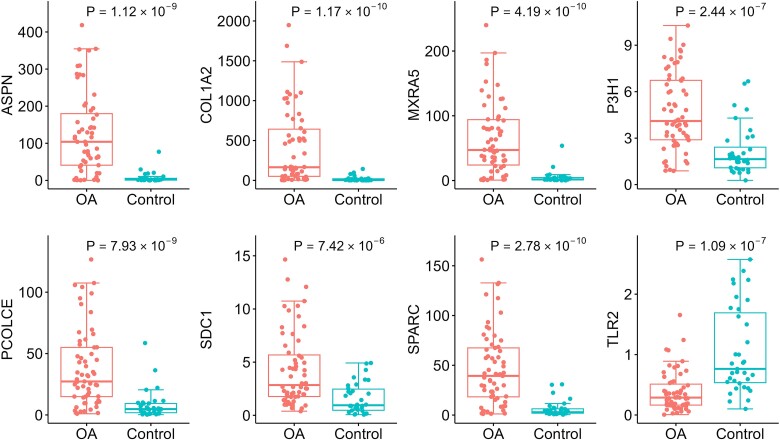
Boxplots of the expression levels of the eight hub genes in validation cohort. OA: Osteoarthritis; *ASPN*: Asporin; *COL1A2*: Collagen type I alpha 2 chain; *MXRA5*: Matrix remodeling associated 5; *P3H1*: Prolyl 3-hydroxylase 1; *PCOLCE*: Procollagen C-endopeptidase enhancer; *SDC1*: Syndecan 1; *SPARC*: Secreted protein acidic and cysteine rich; *TLR2*: Toll-like receptor 2.

The expressions levels of the 8 hub genes identified in the discovery cohort were validated by qPCR. Wilcoxon signed-rank test showed that in the validation cohort, the expression levels of *ASPN* (*P* = 1.21 × 10^−9^), *COL1A2* (*P* = 1.17 × 10^−10^), *MXRA5* (*P* = 4.19 × 10^−10^), *P3H1* (*P* = 2.44 × 10^−7^), *PCOLCE* (*P* = 7.93 × 10^−9^), *SDC1* (*P* = 7.42 × 10^−6^), and *SPARC* (*P* = 2.78 × 10^−10^) in OA-affected cartilage were significantly higher than in OA-free cartilage, while the expression level of *TLR2* (*P* = 1.09 × 10^−7^) was significantly higher in the OA-free cartilage than in OA-affected cartilage. qPCR confirmed that the expression levels of all eight hub genes were significantly changed with the same effect directions as in the discovery cohort (Fig. 5).

### Identification of druggable targets

The druggability status of the eight hub genes was checked against the druggable genome database [19]. Out of these eight hub genes, six were present in the druggable genome database. *SDC1* was classified as Tier 1, which was defined as efficacy targets for approved small molecule and biologic therapeutics drug candidates. *COL1A2*, *PCOLCE*, *SPARC*, *ASPN*, and *TLR2* were classified as Tier 3, which was defined as genes encoding secreted or extracellular proteins, proteins with a more distant similarity to approved drug targets, and members of key druggable gene families that are not included in Tiers 1 or 2.

## Discussion

We examined the transcriptomic profiles of hip cartilage from patients with hip OA and compared them to those from OA-free individuals. Our results indicated that abnormalities in the formation of the ECM and an imbalance in the proportion of collagen chains may play a role in the development of hip OA. Additionally, we identified *SDC1* as a promising potential target for developing new OA treatments.

Five out of the eight hub genes, *ASPN*, *MXRA5*, *P3H1*, *PCOLCE*, and *SPARC,* were the components of collagen-containing ECM. *ASPN* is a member of the small leucine-rich proteoglycan family and has been identified as a susceptibility gene for OA [20]. *ASPN* promotes cartilage degradation by inhibiting TGF-β mediated chondrogenesis, thereby disrupting normal ECM turnover [21, 22]. *MXRA5* is a protein involved in ECM remodeling and plays a crucial role in maintaining chondrocyte integrity and regeneration [23]. Moreover, N-glycosylation level of *MXRA5* was higher in medial than in lateral subchondral bone in primary knee OA [24]. However, the role of *MXRA5* in hip OA remains unclear. It was believed that *MXRA5* interacted with other ECM proteins to regulate cartilage integrity. Overexpression of *MXRA5* may lead to impaired ECM remodeling and contribute to OA progression. *P3H1* is an enzyme involved in the post-translational modification of collagen, specifically catalyzing the hydroxylation of the carbon-3 position of prolyl residues [25]. *P3H1* mutations can cause recessive osteogenesis imperfecta by forming abnormal collagen [26]. To the best of our knowledge, this was the first study to report increased expression of *P3H1* in OA patients. However, the mechanism of overexpressed *P3H1* in the pathogenesis of OA needs further investigation. *PCOLCE* enhances the activity of procollagen C-proteinases which are essential for the maturation of collagen molecules [27]. *PCOLCE* triggers collagen fibrillogenesis by accelerating procollagen maturation through BMP-1/tolloid-like proteinase [28]. Bone morphogenetic protein 1 (*BMP1*) has been observed to be increased in iliac crest bone in patients with hip OA; we also found that the expression level of *BMP1* was increased in OA-affected cartilage in our discovery cohort (*P* = 6.14 × 10^−5^) [29]. Therefore, dysregulation of the pathway from *PCOLCE* to *BMP1* may cause or accelerate hip OA pathogenesis. *SPARC*, also known as osteonectin, is a matricellular protein that modulates cell matrix interactions and ECM assembly; it binds to collagen and influences its deposition and organization [30]. *SPARC* has been observed to increase in both OA and rheumatoid arthritis [31]. Moreover, articular chondrocyte degeneration is promoted by increased *SPARC* synthesis in rat models [32]. These five hub genes are important to the maintenance and remodeling of the collagen-containing ECM. Their dysregulation can disrupt collagen homeostasis, leading to the degradation of cartilage in OA.

Type II collagen constitutes 90%–95% of the collagen in healthy joint cartilage, while type I collagen is almost absent [33]. However, a 100-fold increase in *COL1A1* mRNA levels was observed in stage III OA compared with stage I OA, suggesting that the expression of *COL1A1* was upregulated in end-stage OA [34]. In the current study, we observed that *COL1A2*, encoding another chain of type I collagen, was the most significantly overexpressed gene in OA-affected cartilage (FC = 23.69, *P* = 1.17 × 10^−10^). In addition, a deletion polymorphism (rs3216902) in *COL1A2* was found to be significantly associated with hip OA in the NL population [35]. rs3216902 is located in the intronic region of *COL1A2*. Given the observation of the upregulation of *COL1A2* in hip OA, this deletion polymorphism may occur in silencers or negative regulatory elements, potentially leading to increased expression of *COL1A2*. To date, no study has reported this locus’s function in gene regulation, and our findings filled this gap. The overexpressed *COL1A2* may reduce the formation of type II collagen by depleting the materials for type II collagen synthesis, thereby affecting cartilage regeneration.

Toll-like receptors are directly involved in the activation and regulation of immune responses [36]. *TLR2*, as a member of the toll-like receptor family, has been reported to be upregulated in OA cartilage in previous studies [37–39]. However, we observed *TLR2* to be significantly downregulated in OA affected cartilage in both the discovery and the validation cohort in the current study, which was consistent with the previous report in which *TLR2* deficiency appeared to reduce chondrocyte anti-oxidative stress and autophagy flux capacity, leading to compromised cartilage homeostasis as a result of chondrocyte dysfunction [40]. Further studies are needed to clarify the role of *TLR2* in OA.

*SDC1* is a member of the heparan sulfate proteoglycan family and mediates cell binding as well as cell signaling [41]. *SDC1* negatively regulates inflammation by inhibiting the strong adhesion of leukocytes to the endothelium [42]. Previous studies found *SDC1* to be transient upregulated in the early stage of cartilage degeneration in the Del1 mouse model, specifically in chondrocytes attempting to repair the damaged cartilage, and it exhibited the highest mRNA level in aging knee joints [43]. Downregulation of *SDC1* by exosomal microRNA-9-5p can alleviate inflammation and cartilage damage in the OA mice model [44]. Furthermore, *SDC1* has been shown to be upregulated by *EZH2,* which lead to cartilage catabolism in chondrocytes models [45]. The opposite effects of *SDC1* in OA may be due to the fact that the effect of *SDC1* in cartilage is context-dependent rather than unidirectional. During the early stages of cartilage degeneration, *SDC1* upregulation may be an attempt by chondrocytes to repair damage. Conversely, constant *SDC1* upregulation could potentially exacerbate cartilage catabolism if the repair process becomes excessive. Therefore, *SDC1* might regulate cartilage repair and degradation processes in a stage-specific manner.

In addition, *SDC1* encodes heparan sulfate proteoglycan that contains one or more covalently linked heparin sulfates [46]. Sulfated forms of heparin sulfate were found to be increased in OA cartilage, which in turn regulated the mRNA levels of catabolic and anabolic markers in mice articular chondrocytes [47]. On the other hand, *SDC1* has similar collagen binding determinants as heparin [48]. Heparin can inhibits inflammation and proliferation of fibroblast-like synoviocytes in rheumatoid arthritis through NF-κB pathway [49]. Therefore, targeting *SDC1* in drug development might presents a promising therapeutic avenue for patients suffering from OA. Indeed, *SDC1* was classified as a Tier 1 druggable target by C. Fiana, *et al* [19]. It has also been proposed that targeting *SDC1* could provide new opportunities in cancer therapy, and several agents targeting *SDC1* have been developed and some of them are already in phase I or II trials in cancer [50]. Thus, it would be interesting to examine the efficacy of these agents in OA.

There are limitations in the study. Firstly, the chondrocyte content in cartilage is low, and all of our OA participants were at end-stage, which means that the cartilage was severely worn, and some patients had insufficient remaining cartilage for RNA extraction. Furthermore. due to ethical reasons, OA-free cartilage could only be obtained from non-pathological fractures patients, which limited the sample size and introduced the potential confounding variable from the fracture injure when performing transcriptomic profiling on fracture patients. Despite this, our results were validated in an independent cohort, indicating that the findings were robust. Secondly, we only validated the gene expression levels but could not determine the causal relationships between DEGs and OA or their roles in OA pathogenesis, both of which require further functional studies. Lastly, the NL population has a unique genetic architecture, which might limit the generalizability of our findings to other populations.

In conclusion, this study presented a comprehensive analysis of the transcriptomic profile of primary hip OA. Through our investigation, 179 DEGs were identified, and eight hub genes were validated. While several of these hub genes have been reported in previous studies, *P3H1* was a novel gene associated with hip OA. Furthermore, we identified *SDC1* as a promising target for developing OA drugs. Together, these findings helped us better understand the molecular mechanisms that drive OA and may assist in developing targeted treatment strategies.

## Materials and Methods

### Patients and samples

This study was part of the Newfoundland Osteoarthritis Study (NFOAS). Study participants were recruited between November 2011 and September 2017 at St Clare’s Mercy Hospital and Health Sciences Centre General Hospital in St John’s, NL, Canada. OA patients in the current study underwent total hip replacement (THR) due to primary hip OA. The diagnosis of OA followed the American College of Rheumatology’s clinical diagnostic criteria for OA [51] with confirmation from the attending orthopedic surgeon and the pathology reports of the femoral heads removed during surgery. OA-free controls underwent bipolar hemiarthroplasty due to non-pathological fractures of the femoral neck and did not have evidence of hip OA based on their medical records, which was further confirmed by pathological examination of the resected joint.

Cartilage samples were obtained from removed joints during the surgery, stored in cryogenic vials, snap-frozen, and then stored in liquid nitrogen (LN_2_). 150 ~ 200 mg frozen cartilage sample was transferred to the homogenizing cylinder together with 1 ml TRIzol Reagent (Thermo Fisher, Waltham, U.S.A) and 150 μl guanidine thiocyanate (Sigma-Aldrich, St. Louis, U.S.A) and homogenized in LN_2_ using a cryogenic mill (Spex Freezer Mill, model 6770, Metuchen, U.S.A) with the following procedure: 10 min pre-cooling, and then 3 cycles of 1 min grinding at maximum frequency with 3 min for cooling between grinding cycles. The sample was then transferred to a 50 ml centrifuge tube and thawed at room temperature (RT), and then incubated for another 5 min after sample has reached RT. 250 μl of chloroform (Thermo Fisher, Waltham, U.S.A) was added to the homogenate which was then transferred to new 2 ml RNase-free tube and shaken vigorously for 15 s to mix thoroughly. The mixture was incubated at RT for 2 ~ 3 min and then centrifuged at 12 000 × *g* for 15 min at 4°C. After centrifugation, the sample was separated into 3 phases: the aqueous phase containing RNA, and the interphase and organic phase containing DNA. The aqueous phase was carefully transferred to a new 2 ml RNase-free tube and used for extracting total RNA by RNeasy Mini Kit (Qiagen, Hilden, Germany) following manufacturer’s standard protocol, which was then stored in −80°C freezers until experiments. RNA samples were randomly separated into two cohorts. Samples in the discovery cohort was used for RNA sequencing/microarray assays, while the remaining samples were used to validate the discovery results.

Ethics approval for the current study was received from the Health Research Ethics Authority of Newfoundland and Labrador (HREB #2011.311). All study participants provided written informed consent for their participation.

### Transcriptomics assay

The quality control (QC) filtering of RNA samples was performed using BioAnalyzer or TapeStation (Agilent Technologies, Waldbronn, Germany). Following the criteria set by Genome Québec (https://www.genomequebec.com), only RNA samples with concentrations > 30 ng/μl and RNA integrity number (RIN) > 6.5 were used for RNA-sequencing (RNA-Seq). The stranded sequencing library was prepared using NEB Directional RNA Library Prep Kit for Illumina (New England Biolabs, Ipswich, U.S.A). RNA was sequenced using the Illumina NovaSeq 6000 S4 PE100 platform (Illumina, San Diego, U.S.A). Raw read counts were normalized by counts *per* million (CPM) method and used in the subsequent analysis [52]. RNA samples with concentrations > 65 ng/μl and RNA integrity number equivalent (RINe) > 7 were profiled using Affymatrix Human Clariom D Array (Affymatrix, Santa Clara, United States) at The Centre for Applied Genomics (https://www.tcag.ca). Three samples were assayed using both methods to assess the reliability of combining data from the two approaches.

### Identification of hub genes

Gene Ontology (GO) enrichment analysis including biological process (BP), cellular component (CC), and molecular function (MF) and Kyoto Encyclopedia of Genes and Genomes (KEGG) enrichment analyses were applied toDEGs to identify the potential biological pathways (*P* < 0.05). The Search Tool for the Retrieval of Interacting Genes/Proteins (STRING) online database (http://string-db.org Version:12.0) was used to build a Protein–Protein interaction (PPI) network and identify the potential interactions between DEGs with minimum interaction score ≥ 0.4 (medium confidence) [53]. Cytoscape 3.10.0 and the CytoHubba plug-in were used to visualize the interaction network and identify the hub genes by estimating betweenness, closeness, degree, Density of Maximum Neighborhood Component (DMNC), Maximal Clique Centrality (MCC) and Maximum Neighborhood Component (MNC) scores [54]. Druggable genome database was used to evaluate the druggability of genes [19].

### Validation of the identified hub genes

For the independent validation cohort, complementary DNA (cDNA) was synthesized using SuperScript IV VILO Master Mix (Thermo Fisher, Waltham, U.S.A). QC of cDNA synthesis was performed by quantitative polymerase chain reaction (qPCR) using no-template controls. Primers were designed using the NCBI Primer-BLAST tool, and then validated by running a standard curve with a five-point serial dilution of pooled cDNA samples. Glyceraldehyde-3-phosphate dehydrogenase (*GAPDH*) was used as an internal reference gene for data normalization. The details of the primers for the eight hub genes were provided in Table 2. qPCR was performed in triplicate using 5 μl of diluted cDNA template, 10 μl Power SYBR® Green PCR Master Mix (Applied Biosystems, Waltham, U.S.A.), and 0.4 μl of 10 uM forward and reverse primers each in a final volume of 20 μl on an Applied Biosystems ViiA 7 system. The cycling conditions employed were as follows: 95°C for 10 min, 95°C for 15 s and 60°C for 1 min, repeated in 40 cycles, followed by melt-curve analysis and agarose gel validation. One of the control samples was selected as the calibrator, and the relative quantification (RQ) of target gene mRNA levels was calculated as the fold change relative to the calibrator using the Livak method [55].

**Table 2:** Primers used in qPCR experiments.

	**Primer sequence (5′ > 3′)**	**Product size**
*P3H1* forward primer	5′-ACTGCCATCGAAGAGGTCCA-3′	107 bp
*P3H1* reverse primer	5′-GGGGGCTCTTTGACACACAC-3′	
*PCOLCE* forward primer	5′-CCCTGAGGATGACGACGGAT-3′	95 bp
*PCOLCE* reverse primer	5′-GCAAAATTGGTGCTCAGTGCC-3′	
*MXRA5* forward primer	5′-TCGACGCGCTCTTCAGTTTTG-3′	54 bp
*MXRA5* reverse primer	5′-GGGTCCCATTGGCAAACACC-3′	
*ASPN* forward primer	5′-ACAAGAGAGCCAAGAAGCCA-3′	117 bp
*ASPN* reverse primer	5′-TGGGACTGAGGTCAAACCTAAA-3′	
*SPARC* forward primer	5′-AGCACCCCATTGACGGGTA-3′	105 bp
*SPARC* reverse primer	5′-GGTCACAGGTCTCGAAAAAGC-3′	
*COL1A2* forward primer	5′-TGGTCTCGGTGGGAACTTTGC-3′	101 bp
*COL1A2* reverse primer	5′-CTGCACCAGGTGGGCCTCTA-3′	
*SDC1* forward primer	5′-CTCTGGGGAGCAGGACTTCA-3′	97 bp
*SDC1* reverse primer	5′-CTGATCCACTGGGGACTGGT-3′	
*TLR2* forward primer	5′-GGTGTTGCAAGCAGGATCCAA-3′	142 bp
*TLR2* reverse primer	5′-TGTCCAGTGCTTCAACCCAC-3′	
*GAPDH* forward primer	5′-TCGCCCCACTTGATTTTGG-3′	106 bp
*GAPDH* reverse primer	5′-GCAAATTCCATGGCACCGT-3′	

qPCR: quantitative polymerase chain reaction; P3H1: Prolyl 3-Hydroxylase 1; PCOLCE: Procollagen C-endopeptidase enhancer; MXRA5: Matrix remodeling associated 5; ASPN: Asporin; SPARC: Secreted protein acidic and cysteine rich; COL1A2: Collagen type I alpha 2 chain; SDC1: Syndecan 1; TLR2: Toll like receptor 2; GAPDH: Glyceraldehyde 3-phosphate dehydrogenase.

### Statistical analysis

Wilcoxon signed-rank test or *Chi*-squared test was used to compare the characteristics between OA-free controls and hip OA patients. Genes expressed in less than 80% of samples were excluded from further analysis. The Wilcoxon signed-rank test was used to compare the concordance between the results of RNA-Seq and microarray assay of the three samples analyzed using both methods, and to identify the DEGs between the two study groups. Bonferroni correction was used to adjust for multiple testing. For the discovery cohort, the significance level was set at ⍺ = 2.97 × 10^−6^ to correct for multiple testing for 16 831 genes. For the validation cohort, the significance level was set at ⍺ = 6.25 × 10^−3^ to correct for multiple testing for eight hub genes. All statistical analyses were performed in R version 4.3.2 with ggplot2 [56], enrichplot [57], DOSE [58], and clusterProfiler [59] packages.

## Supplementary Material

Supplementary_materials_ddae200
